# Selective autonomic stimulation of the AV node fat pad to control rapid post-operative atrial arrhythmias

**DOI:** 10.1371/journal.pone.0183804

**Published:** 2017-09-13

**Authors:** Marco A. Mercader, Dingchao He, Aditya C. Sharma, Mark C. Marchitto, Gregory Trachiotis, Gene A. Bornzin, Richard Jonas, Jeffrey P. Moak

**Affiliations:** 1 Department of Medicine, Division of Cardiology, George Washington University, Washington, D.C., United States of America; 2 Department of Surgery, Division of Cardiac Surgery, Children’s National Health System, Washington, D.C., United States of America; 3 School of Medicine, George Washington University, Washington, D.C., United States of America; 4 Department of Surgery, Division of Cardiothoracic Surgery, George Washington University, Washington, D.C., United States of America; 5 Implantable Electronic Systems Division (IESD), St. Jude Medical, Sylmar, California, United States of America; 6 Department of Pediatrics, Division of Cardiology, Children’s National Health System, Washington, D.C., United States of America; University of Minnesota, UNITED STATES

## Abstract

Junctional ectopic tachycardia (JET) and atrial fibrillation (AF) occur in patients recovering from open-heart surgery (OHS). Pharmacologic treatment is used for the control of post-operative atrial arrhythmias (POAA), but is associated with side effects. There is a need for a reversible, modulated solution to rate control. We propose a non-pharmacologic technique that can modulate AV nodal conduction in a selective fashion. Ten mongrel dogs underwent OHS. Stimulation of the anterior right (AR) and inferior right (IR) fat pad (FP) was done using a 7-pole electrode. The IR was more effective in slowing the ventricular rate (VR) to AF (52 +/- 20 vs. 15 +/- 10%, p = 0.003) and JET (12 +/- 7 vs. 0 +/- 0%, p = 0.02). Selective site stimulation within a FP region could augment the effect of stimulation during AF (57 +/- 20% (maximum effect) vs. 0 +/- 0% (minimum effect), p<0.001). FP stimulation at increasing stimulation voltage (SV) demonstrated a voltage-dependent effect (8 +/- 14% (low V) vs. 63 +/- 17 (high V) %, p<0.001). In summary, AV node fat pad stimulation had a selective effect on the AV node by decreasing AV nodal conduction, with little effect on atrial activity.

## Introduction

Open-heart surgery patients are at risk of developing supraventricular arrhythmias including atrial tachycardia (AT), junctional ectopic tachycardia (JET), and atrial fibrillation (AF). Adult patients develop post-operative AF in 33.7% of open heart surgeries in the United States [1]. In children, post-operative atrial arrhythmias occur in 20–30% of patients (JET—10–15%, and AT—5–10%) [2]. These arrhythmias result in low cardiac output and hypotension, thus compromising recovery from surgery [3].

Currently, we depend on the administration of intravenous medications or external cardioversion to control these post-operative arrhythmias. Medication administration can result in adverse effects, in some drug studies up to 50–75% patients have adverse effects [4]. External cardioversion involves delivery of a high voltage electrical discharge to the entire heart that may result in depression of cardiac function, particularly impacting contractility of the atria. Atrial “stunning” is a well documented phenonemon following DC cardioversion of AF associated with thrombus formation within the atria [5].

There is a need for developing non-pharmacologic ways to control heart rate. Prior animal studies demonstrated that stimulation of parasympathetic nerve fibers located on the epicardial fat pad induced a decrease in mean ventricular rate during AF [6,7,8]. Further studies conducted in mongrel dogs by Schauerte and colleagues showed that electrical stimulation of parasympathetic nerves in the inferior vena cava (IVC) resulted in negative dromotropic effects during AF [9]. Rossi et al have tested epicardial vagal fat pad stimulation in humans for control of post operative atrial fibrillation with using a bipolar heart wire near the anterior fat pad region [10].

We have previously developed an animal model of JET and AF to serve as the basis to assess new treatments for post-operative arrhythmias [11]. We have also shown, in a canine model, that stimulation of the AV node fat pads can control the heart rate during junctional ectopic tachycardia and atrial fibrillation [12].

The purpose of our experiments were to test the following aims: 1) anterior right fat pad stimulation is less effective than inferior right AV nodal fat pad stimulation in slowing the ventricular response during atrial arrhythmias, 2) selective site stimulation with the AV nodal fat region might result in variable effects on AV nodal conduction, and 3) a relationship would exist between the intensity of parasympathetic neurostimulation (voltage) and either slowing or termination of junctional ectopic tachycardia and atrial fibrillation.

## Methods

### Experimental preparation

All animals received humane care in compliance with the Guide for the Care and Use of Laboratory Animals and approved animal protocols at The George Washington University. Animals were bred for research purposes at an outside private facility and purchased by WISE Animal Laboratory at George Washington University, specifically and only for our experiments. Animals were housed for 3 days; only one canine was housed at a time, they were all cared at our animal facility with standard diet and care. Ten mongrel dogs, age 9.3 ± 3.15 months and weighing 21.4 ± 2.1 kg, 6 female / 4 male, underwent open-heart surgery replicating Tetralogy of Fallot repair. Tetralogy repair is the most commonly associated with JET. Therefore, the studies attempt to mimic a Tetralogy repair by performing a VSD closure and RVOT patch augmentation.

The canines were brought to the Washington Institute of Surgical Engineering surgical laboratory after fasting for 24 hours. Anesthetic induction followed premedication with 6–13 mg/kg IM tiletamine/zolazepam (Telazol). The animals were intubated with a 7.5 ET tube and ventilated with room air supplemented with oxygen to maintain normal oxygen saturation using a respirator. Throughout the experiment, oxygen saturation was kept greater than 95%. The body temperature was monitored with a rectal probe and kept at 38 degrees Celsius using a temperature-controlled, heated water blanket. Anesthesia was maintained with 1% to 3% isoflurane, supplemented with midazolam (0.05–0.1 mg/kg) and sufentanil (loading dose of 0.5 ug/kg/min and continuous intravenous drip of (1–2 ug/kg/hr) throughout the experiment.

Normal saline at 100 ml/hr was infused to replace spontaneous fluid and blood losses. The arterial blood gases and electrolytes were monitored using an I-STAT analyzer and EG7+test cartridges (Abbott Laboratories, Abbott Park, III).

Using surgical practice, femoral artery and venous cannulation were performed using the Seldinger technique for monitoring arterial blood pressure, laboratories and gases and central venous pressure, respectively. Two body surface electrocardiographic leads (II and III) were monitored for heart rate and cardiac rhythm assessment. The signals were amplified, digitized, and displayed using an AD Instruments PowerLab 8 data acquisition system with LabChart Pro, version 7.1 (AD Instruments, Colorado Springs, Colo).

### Surgical methodology

**Fig 1:**
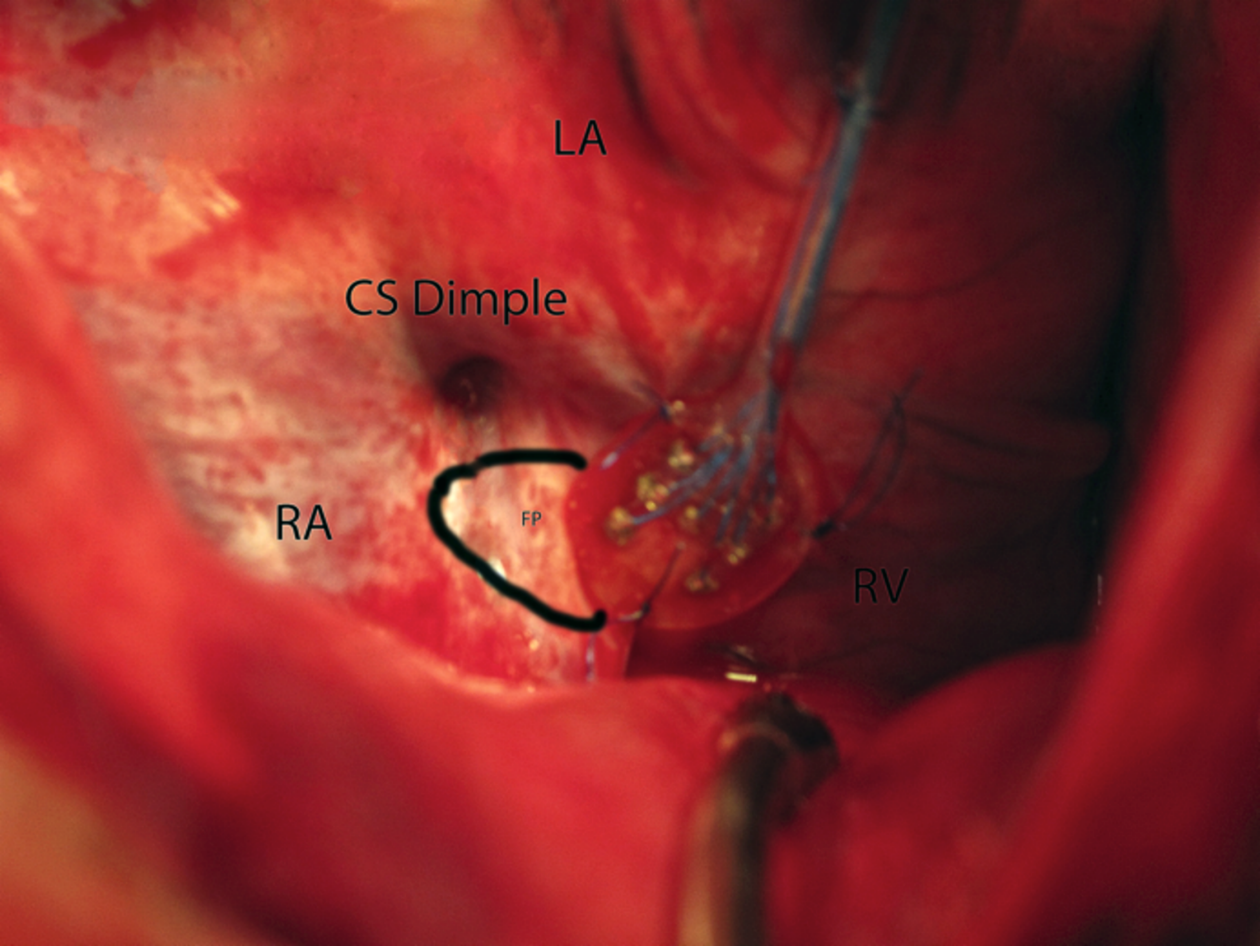
Anatomic relation of seven-pole plaque electrode. Posterior view of right atrium (RA), inferior right fat pad area (FP), and implantation site of seven-pole plaque electrode as seen intra-operatively. The anatomical relation among the left atrium (LA), Coronary Sinus (CS) dimple and right ventricle (RV) is also visible.

Using surgical technique, a median sternotomy was made and the pericardium was opened. After creation of a pericardial cradle, four pacing wires were sewn to the left atrial appendage (two bipolar pairs–one bipolar pair for recording atrial activity and one bipolar pair for pacing) and two pacing wires were sewn to the right ventricle (one bipolar pair to be used for recording ventricular activity). A custom designed plaque electrode was sewn to both the anterior right–lateral and right posterior-inferior fat pads, shown in Fig 1. The distance between the stimulation poles was 2 mm and each electrode was 1 mm in diameter. Fat pad identification was done by the surgeon using a visual assessment were most of the fat tissue was present and then tested for effect. The right and left vagus nerve were exposed and isolated in the upper mediastinum to allow for vagal nerve stimulation. Nerve stimulation (Grass-Telefactor S88X stimulator) was performed to assess for adequacy of plaque electrode placement.

After completion of the baseline fat pad stimulation protocol, cardiopulmonary bypass was instituted to facilitate induction of JET and AF. Heparin (200 units/kg, IV) was administered for a goal activated clotting time above 400 seconds. The superior and inferior vena cava was cannulated using a 20–32 Fr. venous cannula, and the aorta was cannulated using a 10–14 Fr. arterial cannula. Cardiopulmonary bypass was initiated using a Sarns S3 machine and membrane oxygenator to complete the circuit. The circuit was primed with a crystalloid solution. Arterial blood samples were obtained from an arterial catheter every 30 minutes.

Once on stable cardiopulmonary bypass, replication of a non-transannular approach to tetralogy of Fallot repair was performed following cardioplegia administration and induction of hypothermia to 30–32°C, as is performed in surgical repair of infants with congenital heart disease. These steps involve: 1) surgical exploration of the right atrium and right ventricle, 2) stretch of the tricuspid valve, 3) suturing a Gortex patch in the peri-membranous region of the right ventricle using pledgeted sutures, and 4) performing a non-transannular right ventricular outflow patch. Aortic cross clamp duration was between 30–60 minutes, and cardiopulmonary bypass time exceeded 120 minutes.

The canine was then weaned from cardiopulmonary bypass after rewarming (normothermia) on dopamine (5–15 mcg/kg/min) and isoproterenol (0.01–0.1 mcg/kg/min). Protamine was administered to facilitate hemostasis. If JET did not spontaneously happen, the sinus node region was crushed with a clamp to produce sinus node dysfunction. If AF occurred during JET induction protocol, then effects on AF were tested. The response of junctional ectopic tachycardia and atrial fibrillation to fat pad neural stimulation was tested using the protocols outlined below.

From previous experiments, 30 Hz was noted to be the optimal frequency and used for all the stimulations this time, therefore no data varying stimulation frequency was acquired or presented in this publication. Voltage was varied until the most effective voltage was found as far as heart rate reduction or change in wenckeback cycle length. Atrial pacing was done in incremental fashion to determine wenckeback cycle length and at rapid rates, atrial fibrillation was induced. When atrial fibrillation was induced, then fat pad pacing was done using multi-pole catheter, varying pair of electrodes to find the one that would provide the maximum effect. For example, pole selection 1–2, 3–4, 5–6, 6–7, 1–3, 1–4, 1–5, 1–6, 1–7 etc. Example of atrial pacing, started at 90% of sinus cycle length, lowered cycle length by 10 msec until AFIB was induced. Fat pad stimulation protocol included variation in pole selection to determine maximum heart rate reduction site. There are 7 poles in the catheter tip. Two are selected at a time, variation of poles produces higher or lower heart rate reductions and then by iteration process the best pole was selected. A constant frequency and stimulation pulse width was used. Neurostimulation was performed continuously for a period of 15–30 seconds, or until the maximum effect was obtained. We did realize that there was a stimulation fatigue effect, but no specific data was collected during this experiments regarding stimulation fatigue.

All animals had normal sinus node function at onset of experiment. Sinus node was crushed in all experiments in order to allow for JET to occur. No formal sinus node testing study was done. Only noting for sinus pauses and bradycardia to occur after crushing sinus node area with a clamp.

### Definition of JET

JET was defined as a supraventricular arrhythmia (same QRS morphology as in sinus rhythm) with no preceding P wave at a rate that exceeded the normal junctional escape rate for age. The pattern of ventriculoatrial (VA) conduction could be 1:1 VA conduction, VA Wenckebach, or dissociated. JET usually exhibited variability in the rate of onset or termination—warm up or cool down—and did not demonstrate sudden onset or termination. The ventricular rate had to be greater than 120 bpm.

### Definition of selective site pacing

The catheter used for stimulation of the fat pad was a plaque “flat shaped” electrode with 7 poles at the distal end; a pair of poles was selected from the proximal end to be able to stimulate a selective region of the fat pad. Using the 7 pole electrodes available, all possible combinations by forming a pair of electrodes for stimulation were used until we found the most effective site. The tip of the catheter is seen in Fig 1. The stimulation effect is minimal at some stimulation poles–for example if the site of stimulation is far from main ganglion cells, or if some cells stimulated have only a weak effect on the AV node. There may also be differential response to different cells stimulated (more vagal vs. more sympathetic effect) at certain sites of the fat pad.

### Statistical analysis

Continuous data were expressed as mean +/- standard deviation. Student’s t test was used for paired sample comparisons. A p value of < 0.05 was considered significant for tests. One way Analysis of Variance was used to analyze the differences among group means and the variation among and between groups. A p value of <0.01 was considered significant for ANOVA test.

## Results

### Inferior right vs. anterior right fat pad stimulation

**Fig 2:**
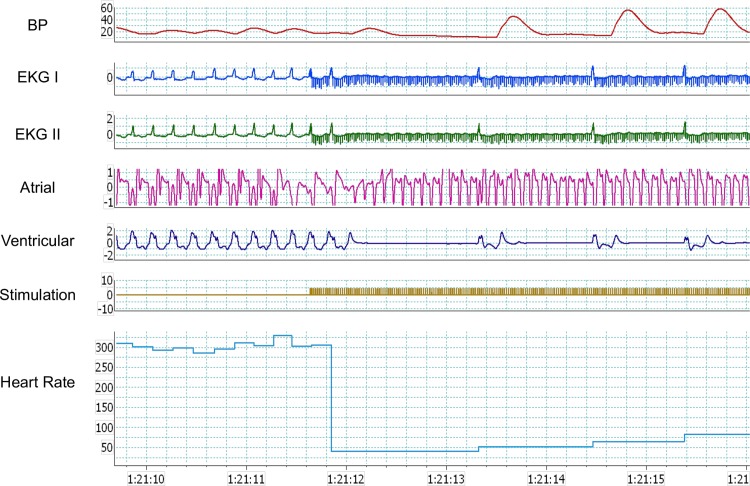
Fat pad stimulation results in slowing of ventricular rate during atrial fibrillation. Fat pad stimulation during atrial fibrillation showing marked slowing of ventricular rate. In sequence from top to bottom, the recordings represent: femoral artery BP tracing, two electrocardiographic leads, atrial and ventricular epicardial electrogram recordings, stimulation artifact documenting FP stimulation, and instantaneous heart rate.

The inferior right FP was more effective in slowing the ventricular rate compared to the anterior right in response to atrial fibrillation in ten animal experiments (52 ± 21 vs. 15 ± 11%, p = 0.003) and JET (12 ± 7 vs. 0 ±0%, p = 0.02.), using a 7-pole plaque electrode sutured to the inferior right fat pad region (Figs 1 and 2).

### Selective site pacing within the fat pad

**Fig 3:**
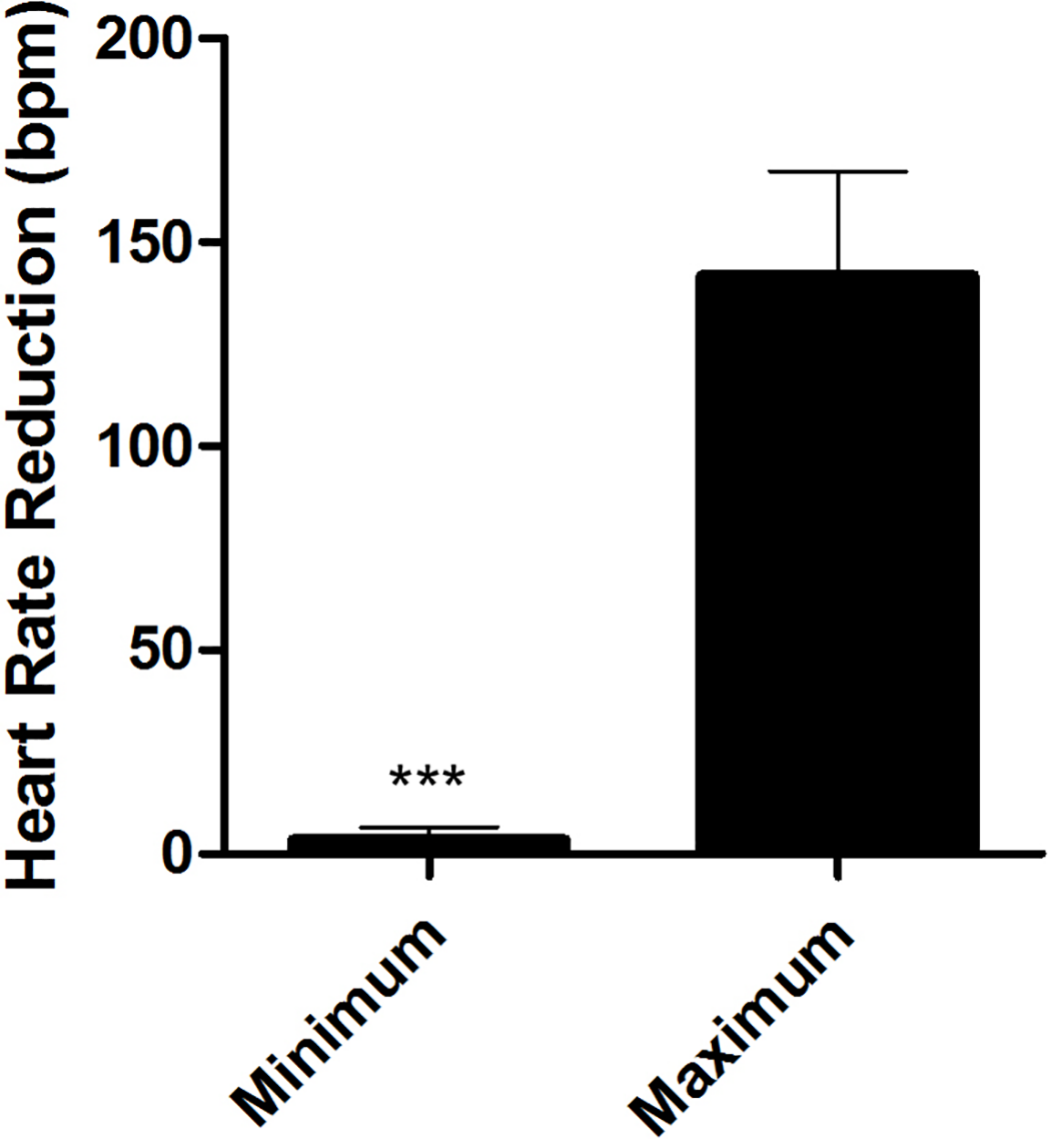
Heart rate reduction following selective site stimulation. Shown are the maximum and minimum heart rate reductions (bpm) at the best and worst selective electrode positions within the seven-pole plaque electrode. ***p<0.001 (two-tailed Student’s t-test)

The effect of stimulation within the inferior right AV nodal fat pad was selective. Selective site stimulation within the inferior right fat pad region demonstrated marked variability on reduction of AV nodal conduction frequency with the effect varying between 0 ± 0% (minimum effect) to 57 ± 21% (maximum effect), p<0.001 (Fig 3).

### Voltage-dependent effect of fat pad stimulation

#### Atrial fibrillation

**Fig 4:**
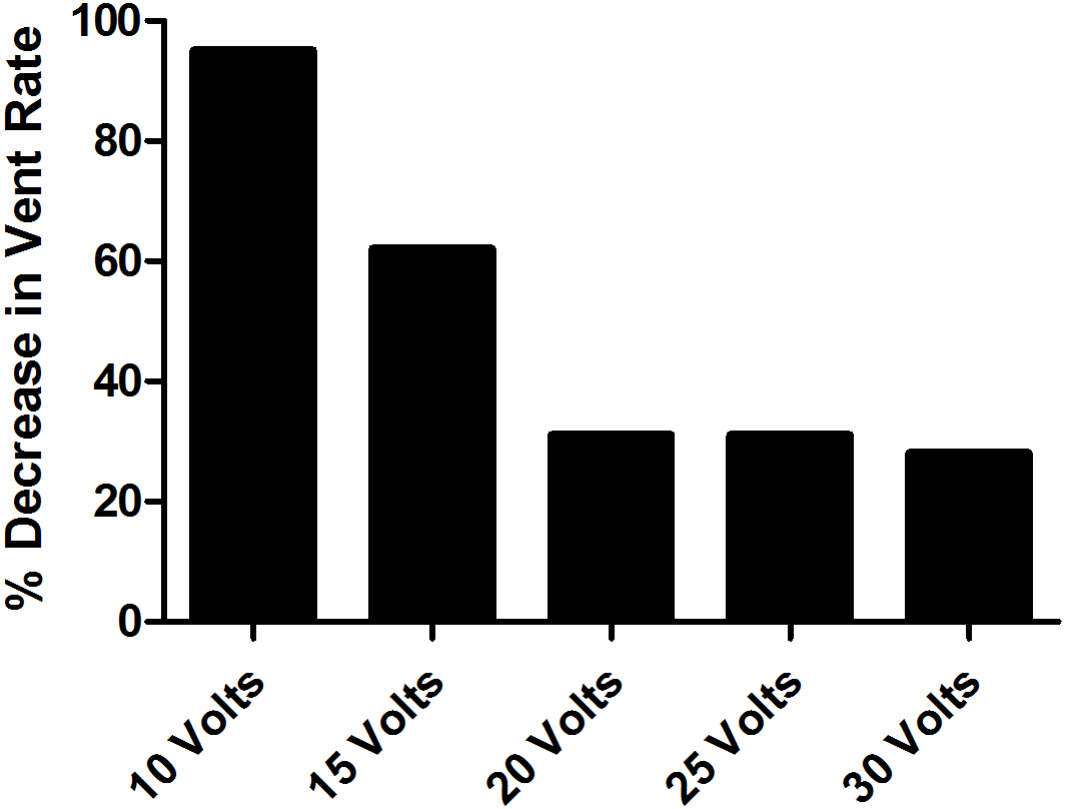
AV Node fat pad stimulation during atrial fibrillation results in voltage-dependent decrease in ventricular rate. Shown is a representative experiment demonstrating AV node fat pad stimulation during atrial fibrillation using the most effective bipolar combination at a constant stimulation frequency and pulse width demonstrating a voltage-dependent decrease in ventricular rate.

**Fig 5:**
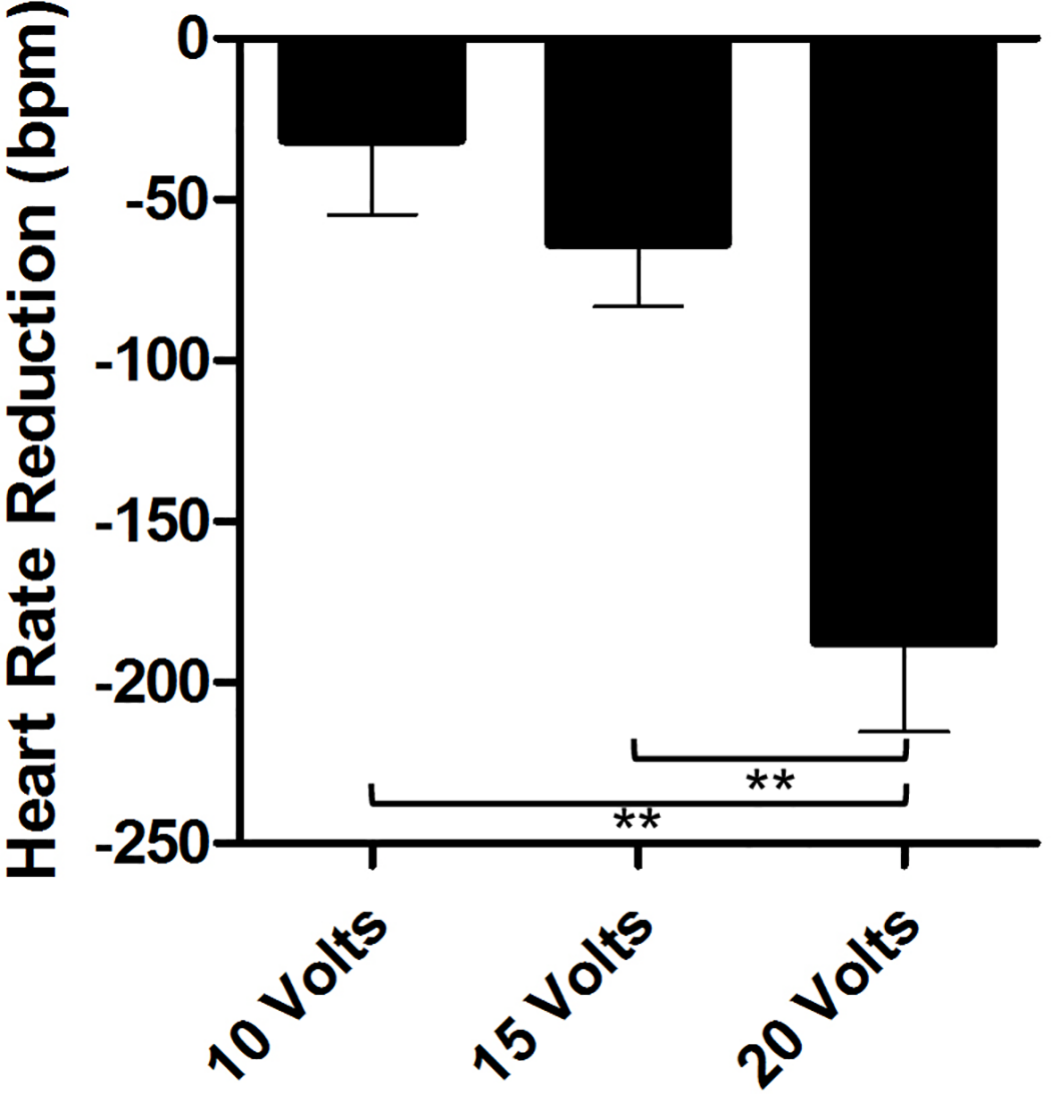
Voltage-dependent response to inferior right fat pad stimulation. Displayed is the relationship between stimulation voltage and absolute heart rate reduction (bpm), n = 10 experiments, illustrating a voltage-dependent response to inferior right fat pad stimulation as voltage amplitude was increased. **p<0.01 (two-tailed Student’s t-test)

Fat pad stimulation at increasing voltage demonstrated a voltage-dependent decrease in ventricular rate (8 ± 14% (low V) vs. 63 ± 17% (high V), p<0.001) (Figs 4 and 5). Using 10, 15 and 20 volts, a voltage-dependent effect was noted with greatest reductions in heart rate observed at higher voltages. The mean heart rate reduction at these voltages was 31 +/- 52 (10 volts), 64 +/- 44 (15 volts) and 187 +/- 62 (20 volts) beats per minute respectively. Using analysis of variance we demonstrated a significant difference between 10 volts compared to 20 volts and between 15 volts compared to 20 volts, p <0.01. Most experiments produced more than twenty episodes of atrial fibrillation.

#### Junctional Ectopic Tachycardia

**Fig 6:**
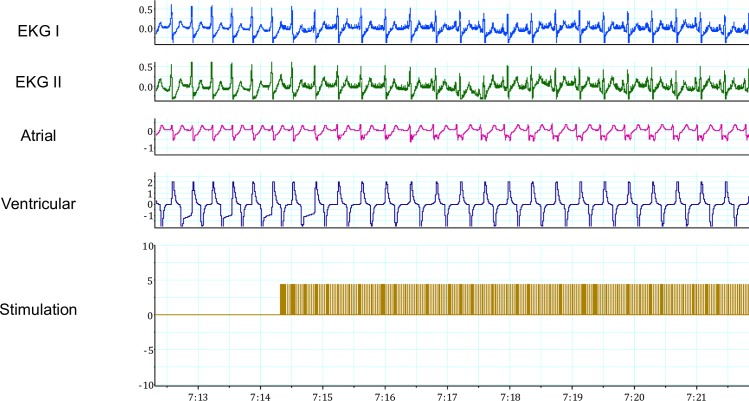
Fat pad stimulation during Junctional Ectopic Tachycardia (JET) at 10 volts. Stimulation during JET resulted in sinus rhythm (SR) with 1:1 atrioventricular (AV) conduction with a normal PR interval. In sequence from top to bottom, the recordings represent: two electrocardiographic leads, atrial and ventricular epicardial electrogram recordings, and stimulation artifact documenting FP stimulation.

**Fig 7:**
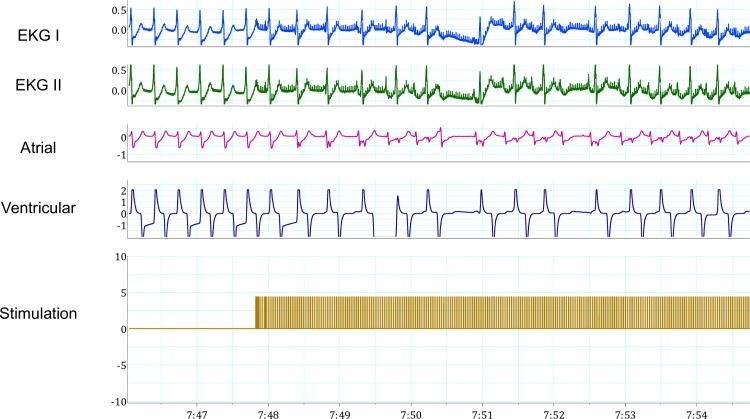
Fat pad stimulation during Junctional Ectopic Tachycardia (JET) at 15 volts. Stimulation during JET resulted in sinus rhythm with second-degree AV block, Mobitz type I AV block. In sequence from top to bottom, the recordings represent: two electrocardiogram leads, atrial and ventricular epicardial electrogram recordings, and stimulation artifact documenting FP stimulation.

**Fig 8:**
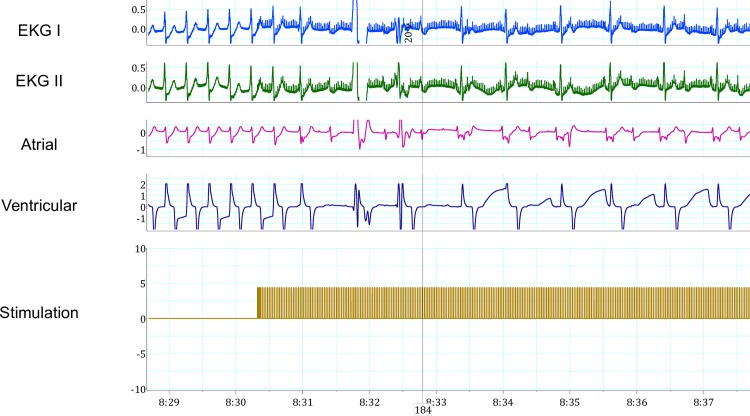
Fat pad stimulation during Junctional Ectopic Tachycardia (JET) at 20 volts. Stimulation during JET resulted in sinus rhythm with 2:1 second degree AV block. In sequence from top to bottom, the recordings represent: two electrocardiogram leads, atrial and ventricular epicardial electrogram recordings, and stimulation artifact documenting FP stimulation.

During JET, inferior right FP stimulation resulted in slowing of JET and increasing AV nodal conduction block (Figs 6–8). There were fewer sustained episodes of JET than atrial fibrillation, on average 3 JET noted per experiments.

## Discussion

### Stimulation of epicardial FP decreases VR during PO JET and AF

The main finding of this study is that the newly designed seven-pole plaque electrode is capable of selectively and effectively stimulate the epicardial fat pads to decrease ventricular rate during PO JET and AF. We tested the catheter using our animal model of PO JET and AF. To achieve slowing of the ventricular rate during atrial arrhythmias, inferior right FP stimulation was more effective than the anterior right FP; this is congruent with previous animal studies but does differ from preliminary data reported in humans by Rossi [10]. No ventricular arrhythmias were induced by the fat pad stimulation. The implantation of the novel catheter was simple, quick and reliable, placed in an easily accessible spot on the epicardial surface of the posterior right atrium at the time of open chest surgery. In clinical practice, the catheter could be placed at the time of surgery and left in place for up to 1-week post operatively to be used as needed.

### Epicardial FP stimulation as a short-term solution following cardiac surgery

Atrial fibrillation can occur in up to 30% of adult patients undergoing coronary bypass grafting (CABG) and up to 50% of adults having combined CABG and valve surgery. The results of the Rate Control versus Rhythm Control for Atrial Fibrillation after Cardiac Surgery study revealed no clinical advantage for the rhythm control strategy [13]. The mean time to onset of postoperative AF was 2.4 days. Adverse medication effects were common in both groups–rate control arm (29%) and rhythm control arm (65%). Twenty-four percent of the patients in the rhythm control group did not complete the course of treatment with amiodarone secondary to adverse drug effects. Most telling was the fact that a very high percent of patients left the hospital in sinus rhythm after an average of 4.3 days after randomization to either treatment group (90% in the rate control arm and 94% in the rhythm control arm). What seems needed in this clinical scenario is a short-term treatment solution as detailed in our study facilitating ventricular heart rate control for a few days, allowing for either spontaneous rhythm conversion to sinus or a decision to electively perform DC cardioversion.

Following open-heart surgery in children, PO JET is the most common serious arrhythmia that occurs in early post-operative period, usually lasting 24–96 hours [14]. The rapid ventricular rate and associated AV dissociation result in low cardiac output. In a previous study, we have shown that magnesium administered at the time of aortic cross clamp release, can decrease the incidence of PO JET from 15.3 to 7.1 [15]. Despite this powerful effect, a significant number of children continue to have PO JET [14]. While intravenous antiarrhythmic medication can facilitate heart rate control, its administration is associated with a high incidence of adverse effects; begging the need for an alternative therapy [15].

Canine models have been used to test chronic AV node vagal stimulation for rate control of atrial fibrillation [16]. Human testing has also shown that epicardial fat pad stimulation is feasible and highly effective technique to decrease mean ventricular rate during atrial fibrillation [10]. Left vagal nerve stimulation has been reported to cause excitotoxicity of the left cervical stellate ganglion [17], which may be avoided by selective local fat pad stimulation. In our experience, only a small section of the visible fat pad can be stimulated to induce significant AV nodal blocking effect. We believe that our catheter design could have significant advantages regarding temporary use in the post-operative period in patients who develop JET or AF to locate the best site for control of ventricular rate. The stimulation of vagal nerves endings at the inferior right fat pad should avoid the decrease in LV function noted on stimulation of the left vagus nerve noted in the past [18].

### Selective nature of fat pad stimulation

The anterior right fat pad has been more selective towards sinus node slowing effects. However, we have seen some evidence of slowing of AV conduction and some of the initial human data obtained by Rossi has also shown some effect on the AV node [10]. The selective nature of fat pad stimulation may correlate with anatomical data showing variable distribution of nerve cell bodies. We noted a consistent voltage dependence effect of fat pad stimulation on heart rate during all experiments.

One important implication of these findings lies in the fact that if the first pair of electrodes selected from the plaque electrode during surgery fails to work or loses its effect, a different pair of electrodes can be selected among them at a later time without having to re-open the chest and reposition the electrode. The inferior right fat pad (AV node fat pad) was more effective in decreasing ventricular rate during AF than the anterior right FP, suggesting its role in control of AV node conduction. The anterior right fat pad had no effect on JET rate compared with the inferior right fat pad, suggesting differences in mechanism of action between the two regions (control of conduction vs. automaticity).

### Study limitations

The catheter was tested in the immediate post-operative period and the results may be different if the same testing were to be performed several days post-surgery, given the changes that can occur from post-operative pericarditis. In particular, the vagal tone may be different under general anesthesia compared to post operative state.

Prior data suggests that autonomic innervation and AV nodal function are similar in canines and humans. However, this has not yet been studied in post-operative subjects. Autonomic vagal tone may be different under general anesthesia in comparison to post-operative patients in different states of sedation.

Finally, the experiments were non-survival and thus we do not have data on fat pad stimulation in the intermediate to long-term post-operative setting.

## Conclusions

The conclusions drawn from our selective site stimulation studies include: 1) the inferior right FP has a greater effect in slowing the ventricular rate during AF and JET, compared to the anterior right FP, 2) there are selective effects from stimulating a specific site within the AV nodal fat pad region and 3) optimizing the voltage output enhanced the efficacy of ventricular rate slowing during post-operative atrial arrhythmias.

Our multipolar electrode design allows for choosing among different sites within the inferior right fat pad allowing for maximazing of ventricular rate slowing. The electrode catheter and pacing device discussed in our study can potentially be used in ventricular rate control in humans at risk for developing post-operative JET and AF following open-heart surgery.

In summary, AV node fat pad stimulation had a selective effect on the AV node by decreasing AV nodal conduction, with little effect on atrial activity. No significant change in the sinus cycle length was observed, thereby showing that the effect is selective to the AV node and AV conduction. AV nodal fat pad stimulation slowed the ventricular rate during PO JET and atrial fibrillation in our young canine open-heart surgery model. These results demonstrate the ability of selective AV nodal fat pad stimulation to not only decrease AV nodal conduction during atrial fibrillation, but also peri-AV nodal automaticity during JET.

## Supporting information

S1 Minimal Data Set This file has the raw data used to generate the figures in the manuscript. (DOCX)
