# Supplementary material for: Selective autonomic stimulation of the AV node fat pad to control rapid post-operative atrial arrhythmias
Source: PLoS One. 2017 Sep 13;12(9):e0183804. doi: 10.1371/journal.pone.0183804 (PMC5597131; doi:10.1371/journal.pone.0183804)
Supplement: S1 Minimal Data Set — (DOCX) [file pone.0183804.s001.docx]

S1 Minimal Data Set

Figure 1: Picture for illustration of fat pad location.

Figure 2: Actual recording during atrial fibrillation and fat pad stimulation to illustrate slowing of heart rate.

Figure 3: Heart rate reduction at worst and best area of stimulation in bpm.

|  |  |  |
| --- | --- | --- |
| Sample #1 | 0 | 220 |
| Sample #2 | 3 | 72 |
| Sample #3 | -2 | 102 |
| Sample #4 | 0 | 155 |
| Sample #5 | 16 | 160 |

Figure 4:

Heart rate at baseline and after 10 volts applied in bpm.

| Sample #1 | 274 | 240 |  |  |
| --- | --- | --- | --- | --- |
| Sample #2 | 266 | 250 |  |  |
| Sample #3 | 270 | 267 |  |  |
| Sample #4 | 248 | 245 |  |  |
|  |  |  |  |  |

Heart rate at baseline and after 15 volts applied in bpm.

| Sample #1 | 272 | 179 |  |  |
| --- | --- | --- | --- | --- |
| Sample #2 | 264 | 167 |  |  |
| Sample #3 | 280 | 188 |  |  |
| Sample #4 | 278 | 158 |  |  |
|  |  |  |  |  |

Heart rate at baseline and after 20 volts applied in bpm.

| Sample #1 | 271 | 86 |  |  |
| --- | --- | --- | --- | --- |
| Sample #2 | 280 | 92 |  |  |
| Sample #3 | 279 | 102 |  |  |
| Sample #4 | 248 | 67 |  |  |
|  |  |  |  |  |

Heart rate at baseline and after 20 volts applied in bpm.

| Sample #1 | 280 | 92 |  |  |
| --- | --- | --- | --- | --- |
| Sample #2 | 269 | 83 |  |  |
| Sample #3 | 286 | 97 |  |  |
| Sample #4 | 280 | 81 |  |  |

Heart rate at baseline and after 30 volts applied in bpm.

| Sample #1 | 271 | 82 |  |  |
| --- | --- | --- | --- | --- |
| Sample #2 | 279 | 73 |  |  |
| Sample #3 | 270 | 93 |  |  |
| Sample #4 | 275 | 71 |  |  |
|  |  |  |  |  |

Figure 5: Heart rate reductions at 10, 15 and 20 volts in bpm.

10 volts

| Sample # 1 | 6 |
| --- | --- |
| Sample # 2 | -94 |
| Sample # 3 | -82 |
| Sample # 4 | 7 |
| Sample # 5 | 6 |
| 15 volts  Sample # 1 | -90 |
| Sample # 2 | -126 |
| Sample # 3 | -20 |
| Sample # 4 | -32 |
| Sample # 5 | -50 |
| 20 volts  Sample # 1 | -136 |
| Sample # 2 | -170 |
| Sample # 3 | -279 |
| Sample # 4 | -220 |
| Sample # 5 | -132 |

Figure 6: Actual recording during JET and fat pad stimulation illustrating conversion to sinus rhythm.

Figure 7: Actual recording during sinus rhythm illustrating Mobitz type I A V block

Figure 8: Actual recording during sinus rhythm illustrating transient 2:1 AV block
